# Could Dietary Supplementation with Different Sources of N-3 Polyunsaturated Fatty Acids Modify the Rabbit Gut Microbiota?

**DOI:** 10.3390/antibiotics11020227

**Published:** 2022-02-10

**Authors:** Giulio Curone, Filippo Biscarini, Elisa Cotozzolo, Laura Menchetti, Alessandro Dal Bosco, Federica Riva, Paola Cremonesi, Stella Agradi, Simona Mattioli, Bianca Castiglioni, Alessia Di Giancamillo, Alice Cartoni Mancinelli, Susanna Draghi, Alda Quattrone, Giulia Collodel, Silvia Clotilde Modina, Cesare Castellini, Gabriele Brecchia

**Affiliations:** 1 Department of Veterinary Medicine, University of Milano, Via dell’Università 6, 26900 Lodi, Italy; giulio.curone@unimi.it (G.C.); federica.riva@unimi.it (F.R.); susanna.draghi@unimi.it (S.D.); silvia.modina@unimi.it (S.C.M.); gabriele.brecchia@unimi.it (G.B.); 2 Institute of Agricultural Biology and Biotechnology (IBBA)–National Research Council (CNR), U.O.S. di Lodi, Via Einstein, 26900 Lodi, Italy; biscarini@ibba.cnr.it (F.B.); cremonesi@ibba.cnr.it (P.C.); castiglioni@ibba.cnr.it (B.C.); 3 Department of Agricultural, Food and Environmental Sciences, University of Perugia, Borgo XX Giugno 74, 06121 Perugia, Italy; elisa.cotozzolo@studenti.unipg.it (E.C.); alessandro.dalbosco@unipg.it (A.D.B.); simona.mattioli@unipg.it (S.M.); alice.cartonimancinelli@unipg.it (A.C.M.); cesare.castellini@unipg.it (C.C.); 4 Department of Agricultural and Food Sciences, University of Bologna, Viale G. Fanin 44, 40137 Bologna, Italy; 5 Department of Biomedical Sciences for Health, University of Milano, Via dell’Università 6, 26900 Lodi, Italy; alessia.digiancamillo@unimi.it; 6 Department of Veterinary Medicine, University of Perugia, Via San Costanzo 4, 06126 Perugia, Italy; alda.quattrone@hotmail.it; 7 Department of Molecular and Developmental Medicine, Policlinico Le Scotte, University of Siena, Viale Bracci, 53100 Siena, Italy; giulia.collodel@unisi.it

**Keywords:** Firmicutes/Bacteroidetes ratio, flax seed, fish oil, polyunsaturated fatty acids, omega 3, caecal fermentation, α-linolenic acid, eicosapentaenoic acid, docosahexaenoic acid, stomach mucosa

## Abstract

The present study evaluated the effects of feed supplemented with two dietary sources of n-3 polyunsaturated fatty acids (PUFAs; fish oil and extruded flaxseed) on the gut microbiota, caecal fermentations, gastrointestinal histology, and histochemistry in rabbits. Fifteen male New Zealand White rabbits were divided into three groups (*n* = 5/group) and fed with different diets from weaning (35 days of age) until slaughtering (90 days of age): C group, fed with a commercial diet; F group, supplemented with 10% of extruded flaxseed; and O group, supplemented with 3.5% of fish oil. At slaughter, the content of the stomach, duodenum, jejunum, ileum, caecum, and colon was collected and analyzed by Next Generation 16S rRNA gene sequencing. Tissue samples of the same tracts were evaluated with histological and histochemical analysis. Ammonia and lactic acid in the caecum were also quantified. Twenty-nine operational taxonomic units (OTUs) were significantly different between groups. Groups receiving n-3 PUFAs supplementation showed an increase in Bacteroidetes and *Lachnospiraceae* in several gastrointestinal tracts, while Bacilli abundance, as well as Firmicutes/Bacteroidetes ratio, were reduced compared to the control group (for all *p* < 0.05). Caecal ammonia was lower in the F than C group (*p* < 0.032), whereas no difference was found for lactic acid. Finally, histological evaluations revealed a mild hemorrhagic infiltration and vessels ectasia in the stomach mucosa of both F and O groups, but no effect of nutritional treatment was evidenced by the histochemical analyses. In conclusion, n-3 PUFAs supplementation could modify the rabbit gut microbiota and fermentation. The increase in beneficial bacterial populations may, at least partially, explain the positive effects of n-3 PUFAs diet supplementation on human and animals’ health, although the appropriate dosage should be established.

## 1. Introduction

The microbial community that colonizes a particular district of the body and lives in a symbiotic relationship with the host is defined as microbiota [1]. The digestive system is normally colonized by a wide range of bacteria, fungi, and viruses [2]. The gut microbiota composition has a strong impact on the general health and digestive functions of the host [3]. The maintenance of a normal microbial community requires a homeostatic equilibrium among the microbes as well as between the microbiota and the host [4]. Generally, the gut microbiota shows a high grade of resilience and resistance to changes, as it is quite stable against different perturbations. Common factors that can lead to a perturbation of the intestinal homeostasis could depend on both environment and host such as the species [5], age [6], genetic factor [7], farm management [8], drug treatment [9], exposure to pathogens [10], and diet [11,12,13]. The disruption of the normal microflora composition and its metabolic activities could alter the microbiota homeostasis resulting in “dysbiosis” and consequently in different diseases, not only localized to the gastrointestinal tract but also at a systemic level [14,15,16].

The rabbit is very sensitive to gastrointestinal diseases, not only when kept as a livestock species but also as a companion animal [17,18]. Diseases of the digestive system are therefore not only a cause of compromising well-being and mortality but also of economic losses. In this context, the gut microbiota assumes considerable importance, as dysbiosis is associated with several disorders such as diarrhea, enterotoxaemia, and gastrointestinal stasis [19]. Rabbits are monogastric, herbivores, and small hindgut-fermenters that use cecotrophy to obtain maximum energy from the digestion and absorption of the nutrients included in plant-based diets. The large intestine represents the fermentation chamber and hosts the highest richness and diversity in commensal bacterial populations compared to other tracts of the digestive system [5]. The bacterial populations that inhabit the different gastrointestinal tracts of rabbits have recently been described [5]. Upcoming challenges include clarifying the regulation mechanisms of the intestinal microbiota that can modulate gastrointestinal functionality, the maturation and activity of the immune system, resistance to diseases, and productive performance [20,21,22]. In this context, particular attention is paid to critical moments of the rabbit’s production cycle such as the post-weaning period and the role that the gut microbiota could play in reducing the enteric disorders, and as a consequence, the use of antibiotics [23,24]. For this reason, new strategies should be found to reduce the incidence of gastrointestinal pathologies that quite often reduce the profitability of rabbit breeding and therefore could have implications for animal welfare and livestock sustainability. Rabbit, finally, is not only a livestock species and a pet but also an animal model to study several physiological processes including digestion as well as productive performance [25,26], reproduction [27,28,29], and immunity [30,31,32]. Recently, the effects of the dietary integration of various natural substances on the reproductive and productive performance of rabbits have been investigated [33,34,35,36,37]. Despite the critical importance of the gut microbiota in rabbit health, only a few studies have investigated the impact of specific diets on the modifications of gut microbial population in this species to date [12,38].

In recent decades, great attention has been paid to evaluate the role of dietary polyunsaturated fatty acids (PUFAs) on the health status as well as on the prevention and treatment of several humans and animals’ diseases [22,39]. Mammals cannot de novo synthesize some n-6 and n-3 PUFAs, in particular, long-chain PUFAs (>20 C). They result from elongations and desaturation of two essential fatty acids: linoleic acid (LA; 18:2n-6) and α-linolenic acid (ALA; 18:3n-3), whose concentrations in the organism only depend on dietary intake [22,39,40]. n-3 PUFAs resulting from eicosapentaenoic acid (EPA, 20: 5n-3) and docosahexaenoic acid (DHA, 22: 6n-3) have anti-inflammatory, antiproliferative, and anti-atherosclerotic activities [41,42,43]. Several studies showed that diets supplemented with nutrient rich in n-3 PUFAs such as flaxseed (source of ALA), fish, and fish oil (source of EPA and DHA) positively influence the immune function, blood pressure, cholesterol and triglycerides levels, as well as cardiovascular function in different animals’ species, including humans [36,44,45,46]. Moreover, there is growing evidence revealing the effects that n-3 PUFAs exert on productive performance, gut physiology, homeostasis, immune tolerance, and gut microbiota establishment and maintenance [47,48,49]. In particular, n-3 PUFAs can influence the gut microbiota in three principal ways: (1) modulating the type and abundance of the microbial community of the intestine [22,39]; (2) reducing the levels of proinflammatory mediators, such as endotoxins (i.e., lipopolysaccharide) and cytokines or promoting the secretion of anti-inflammatory factors [50,51]; and (3) regulating the growth of butyric acid-producing bacteria [20,48]. Butyrate is considered an important source of energy for the intestinal epithelium, maintains intestinal homeostasis, and controls the gene expression, proliferation, differentiation, and apoptosis of the intestinal cells, as well as the inflammatory response [20,52]. However, knowledge of the correlations between n-3 PUFAs and the intestinal microbiota in livestock animals and particularly in rabbits is limited [47,52].

In this study, we hypothesize that n-3 PUFAs-enriched diets influence bacterial richness and diversity of rabbits’ digestive system. The aim was to evaluate the effect of flaxseed and fish oil supplemented diets on the gut microbiota composition in stomach, duodenum, jejunum, ileum, caecum, and colon of rabbits using next-generation 16S rRNA gene sequencing. In addition, histology and histochemistry were performed on the same sections of the digestive system. Finally, lactic acid and ammonia produced by the cecal bacterial fermentations were also quantified.

## 2. Results

### 2.1. Sequencing Results

The microbiota structure of the gastrointestinal tract of Control (C), Flaxseed (F), and Fish oil (O) groups was characterized by a total of 3,230,834, 3,056,689, and 3,201,736 high quality reads (after filtering), respectively, with a mean of about 130,000 ± 55,000 reads per group. The average reads loss due to filtering was 27%.

### 2.2. Taxa Composition of Gastrointestinal Microbiota in Rabbit of Control (C), Flaxseed (F) and Fish Oil (O) Groups

**Figure 1:**
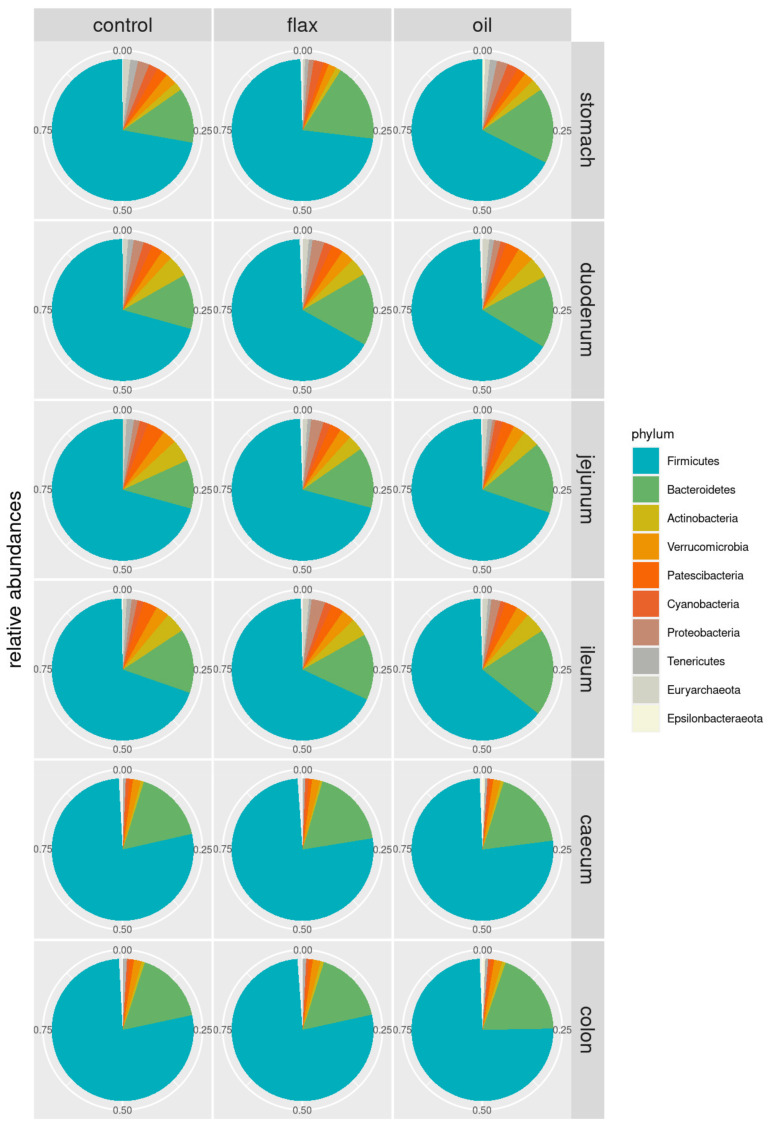
Pie chart of phylum relative abundances along the gastrointestinal tract in control (*n* = 5), flax (supplemented with flaxseed, *n* = 5) and oil (supplemented with fish oil, *n* = 5) rabbits.

**Figure 2:**
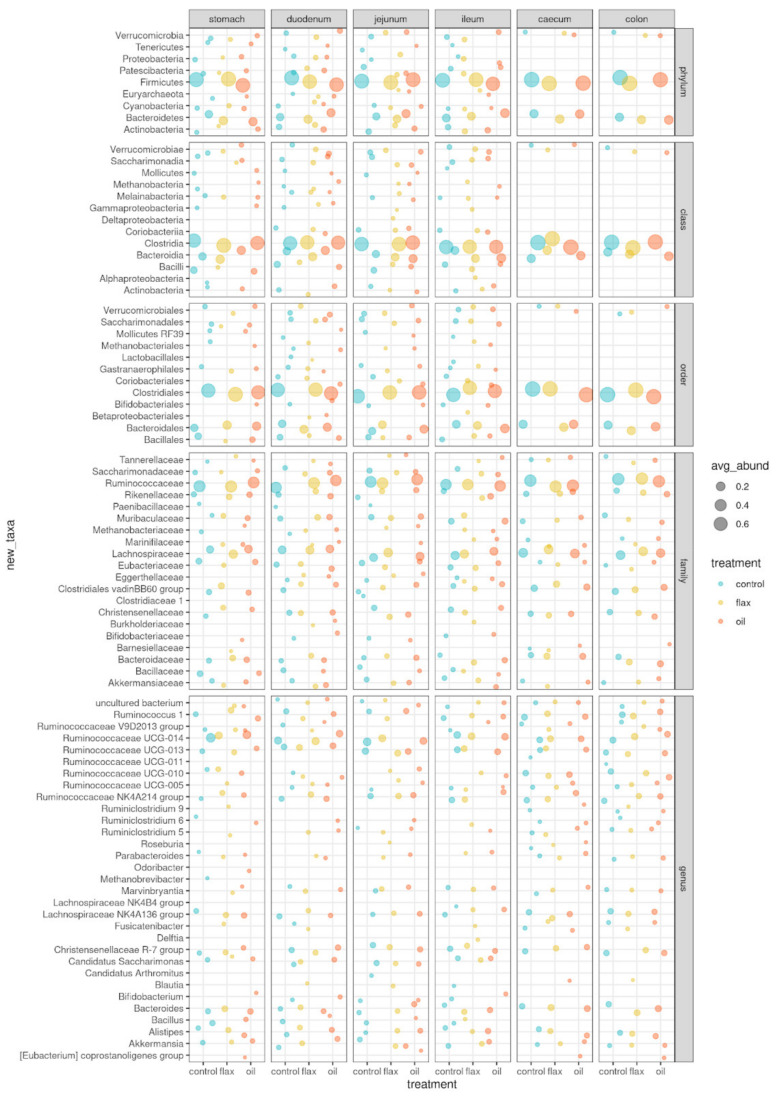
Bubble chart of relative abundances of all taxa (≥1%) in the gut microbiota of the three groups of rabbits, grouped by taxonomic level (from phylum to genus). Control (blue = 5 rabbits), flaxseed (yellow = 5 rabbits) and fish oil (red = 5 rabbits) experimental groups. The size of the bubble is proportional to the relative abundance, with 0.2, 0.4, and 0.6 hallmarks, as shown in the legend.

**Figure 3:**
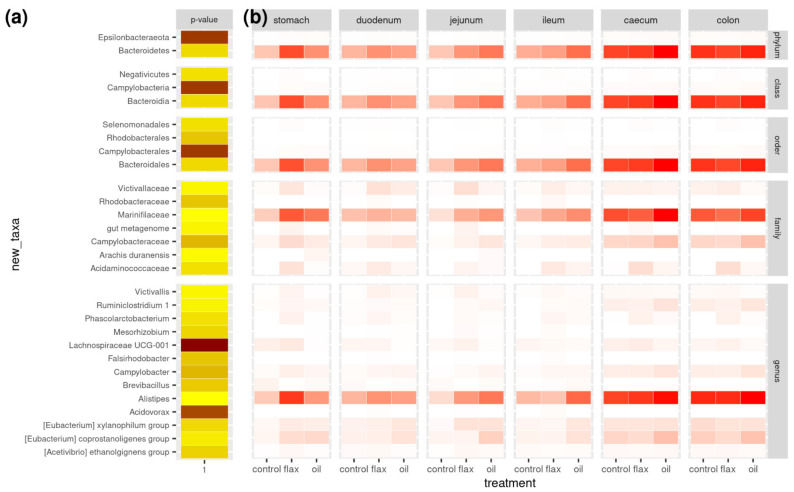
Heatmap of significance and abundance of significantly different taxa. In (**a**), the -log(*p*-value) for the difference between treatments along the gastrointestinal tract is reported (*p*-values are in the range 10–15–0.049, from dark brown to light yellow). In (**b**), the relative abundances of each significant OTUs in the consecutive sections of the gastrointestinal tract of rabbits are shown (darker colors indicate higher abundance). OTUs are grouped per taxonomic level (from phylum to genus). Groups (*n* = 5/group): control and enriched diets supplemented with flaxseed (flax) and fish oil (oil). *p*-values were obtained from a linear model that included the effects of treatment and tissue within rabbit.

Phylum relative abundance distributions in the different gastrointestinal tracts of C, F, and O groups are shown in Figure 1. The main taxa (relative abundance > 1%) detected in the rabbit’s gut microbiota are presented in Figure 2 (and Tables S1 and S2). Without major differences between groups, the most abundant phyla were Firmicutes and Bacteroidetes. Both phyla tended to increase moving from the stomach to the colon; O group showed higher Bacteroidetes levels in the jejunum (16.9%), ileum (19.9%), and colon (19.4%) compared to C group. Bacilli, Bacteroidia, and Clostridia were the most abundant classes, with Bacilli less abundant in the O and, especially, F groups compared to C, in the early portion of the digestive tract (stomach: 8.6%, 1.5%, and 5.8%; duodenum: 7.3%, 2.8%, and 3.9%; jejunum: 5.6%, 2.7%, 2.9% in C, F, and O groups, respectively). The same pattern is reflected in orders: the prevailing orders are Bacillales, Bacteroidales, and Clostridiales, with Bacillales more abundant in C than treated groups (stomach: 8.4%, 1.2%, and 4.9%; duodenum: 5.7%, 1.8%, and 3.3%; jejunum: 4.6%, 1.8%, and 2.7% in C, F, and O groups, respectively). As for families, *Ruminococcaceae* is the most abundant (between 33% and 42%), followed by *Lachnospiraceae* (11%–20%), *Clostridiales vadinBB60 group*, *Muribaculaceae*, *Bacteroidaceae,* and *Bacillaceae* (this latter in C, but not in treated groups). *Lachnospiraceae* were higher in F (from 15.7% to 20.3%, depending on the tissue) and more or less similar in O (from 13.7% to 20.1%) and C (from 11.0% to 20.5%). At the genus level, relative abundances are clearly more spread across genera, with significant differences between treatments in 13 genera, among which *Lachnospiraceae* UCG-001 (*p* < 0.001), *Acidovorax* (*p* < 0.01), *Campylobacter* (*p* < 0.05), and *Brevibacillus* (*p* < 0.05). In total, 29 operational taxonomic units (OTUs) were significantly different between groups: these are reported in Figure 3 and Table S1, together with their relative abundances in addition to the gastrointestinal tract of rabbits. In addition, Tables S2 and S3 show the abundance of the significantly different taxa according with group and gastrointestinal tract.

### 2.3. Firmicutes/Bacteroidetes (F/B) Ratio

**Figure 4:**
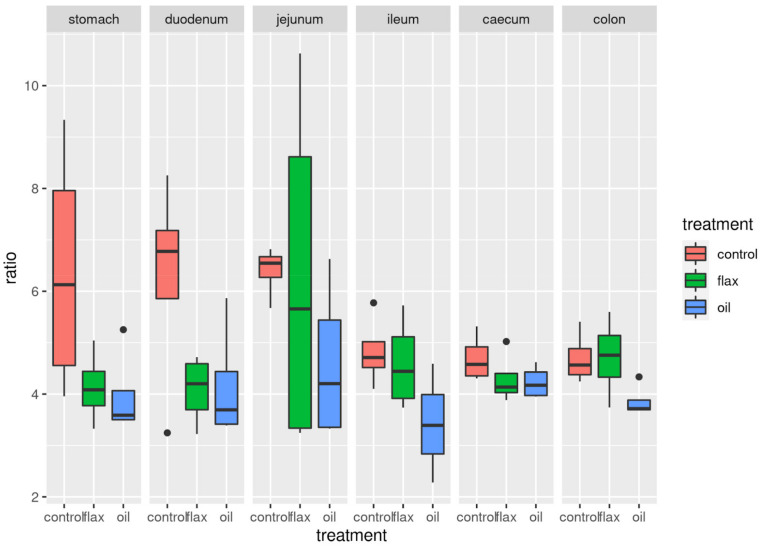
Boxplots of the distribution of F/B ratio (Firmicutes to Bacteroidetes) in the gut microbiota of rabbits, for the three dietary groups (control, and diets supplemented with flaxseed (flax) and fish oil (oil); *n* = 5/group) along the digestive tract.

Regardless of the gastrointestinal tracts, the F/B ratio in the rabbit gut microbiota was consistently higher in the C group compared to F and O groups (in this order; *p* = 0.0014), but with a large individual variation (Figure 4). When correcting for the digestive tract section (Figure 4 and Table S4), the F/B ratio differences between dietary treatments from a linear model that included both tissue and treatments terms were not significant (*p* > 0.05).

### 2.4. Alpha Diversity

**Figure 5:**
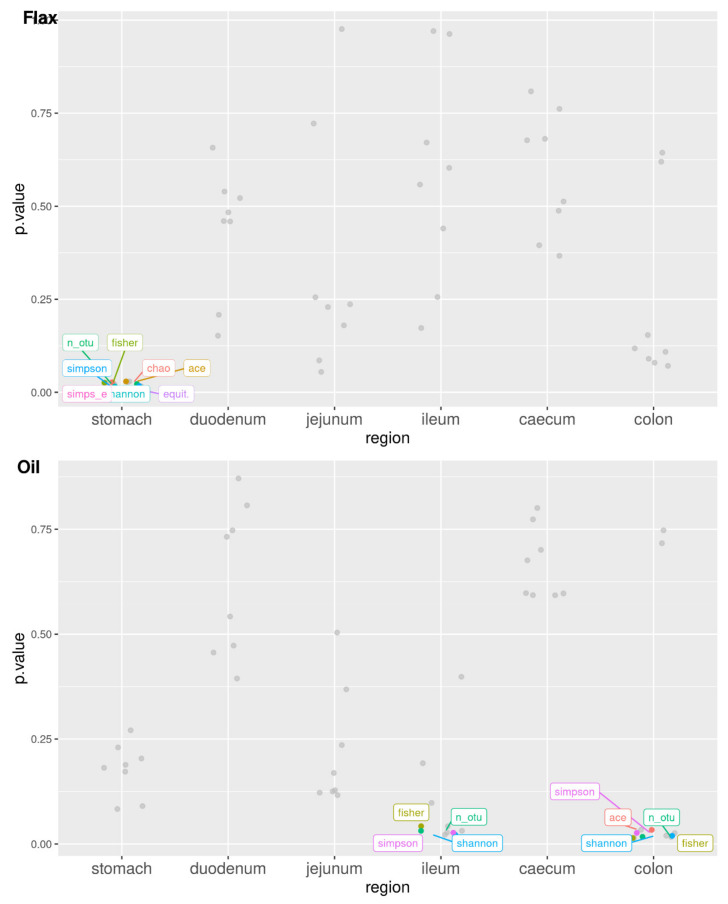
Significance of treatment differences for the alpha diversity indices along the rabbit’s digestive tract (region; threshold: *p* < 0.05) for diets supplemented with flaxseed (flax) and fish oil (oil). Linear model of the form: alpha index = mu + treatment + e for each gastrointestinal tract (tissue) separately.

Table 1 shows the values for the alpha diversity indices calculated from the OTU table in the three experimental groups along the rabbit’s digestive system. As highlighted in Figure 5 and Figure S1, the F group is significantly different from the C group in the stomach, while the O group is significantly different from C further down in the gastrointestinal tract, in the ileum and colon.

### 2.5. Beta Diversity

**Figure 6:**
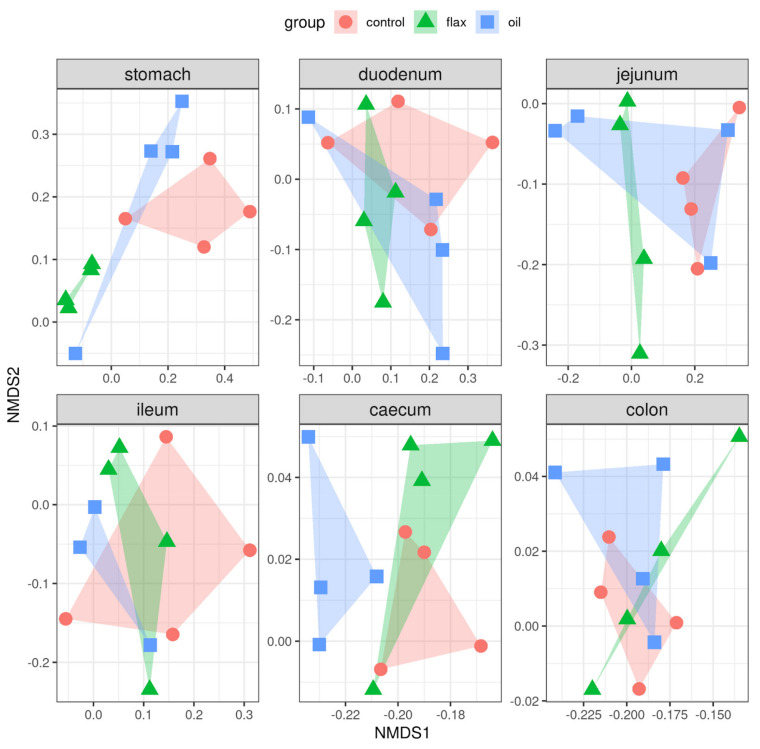
MDS plot of Bray–Curtis distances based on the filtered and normalized OTU table. ClusTable 5. group) along the rabbits’ digestive tract. *p*-values from permutational analysis of variance (PERMANOVA) with 999 permutations.

As for Bray–Curtis distances, from the first two dimensions of the MDS plot, there appears to be some clustering of samples by treatment in the stomach, ileum, and caecum (Figure 6).

Based on PERMANOVA results, only the differences in the stomach turned out to be significant (*p* = 0.010; Table S5), which may well be a consequence of the limited sample size (11–12 samples per section of the digestive tract).

### 2.6. Lactic Acid and Ammonia Quantification in Cecal Content

Lactic acid quantification showed no statistically significant differences among groups, while there was a significant difference among C and F groups for the ammonia concentration (*p* = 0.032), as reported in Table 2.

### 2.7. Histology and Histochemistry

**Figure 7:**
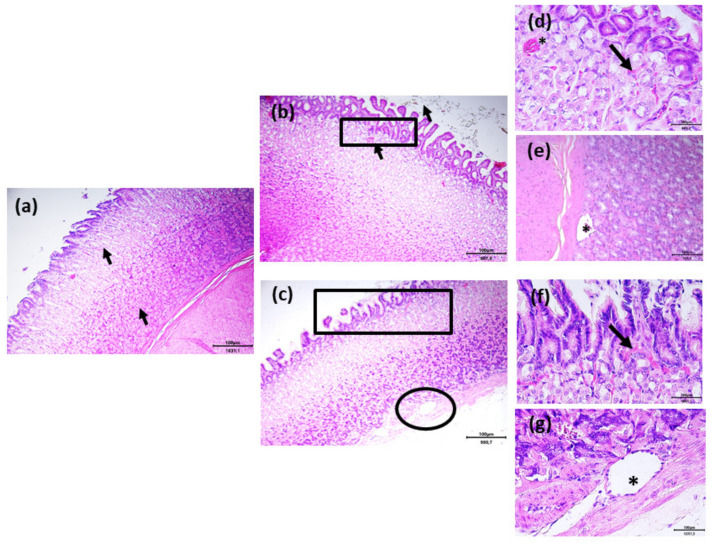
HE haematoxylin/eosin staining. The fundic mucosa of the stomach is regularly organized in control (**a**), group supplemented with flaxseed (**b**), and with fish oil (**c**). Blood cell infiltrates ((**d**) for group supplemented with flaxseed and (**f)** for group supplemented with fish oil) as well as enlarged vessels ((**e**) for group supplemented with flaxseed and (**g**) for group supplemented with fish oil) were present in treated animals. Proper scale bar is located in each figure. For the interpretation of the arrow and the asterisk, see the text.

The structure of the intestinal tracts was always anatomically normal and comparable in all the experimental groups, except for the fundic mucosa of the stomach. Fundic mucosa of the control group showed a normal organization with properly called gastric glands immersed in the lamina propria (Figure 7a, arrows). As the control group, F group revealed a structurally normal mucosa of the secreting lining epithelium and the gastric glands, although it was possible to notice dilated vessels in the lamina propria towards the lumen (Figure 7b, arrow). At higher magnification (rectangular box), it was possible to observe apical ectatic vessels of the lamina propria (Figure 7e, asterisk) and a small quantity of hemorrhagic infiltrate in the connective tissue lamina propria (Figure 7d, arrow). Finally, the O group showed a normal mucosa, and in this case, dilated vessels were also observed in the lamina propria towards the lumen compared to control (Figure 7c). At higher magnification, it was possible to observe a small quantity of hemorrhagic infiltrate in the lamina propria (rectangular box, Figure 7f, arrow) and basal ectatic vessels (circular box, Figure 7g, asterisk).

**Figure 8:**
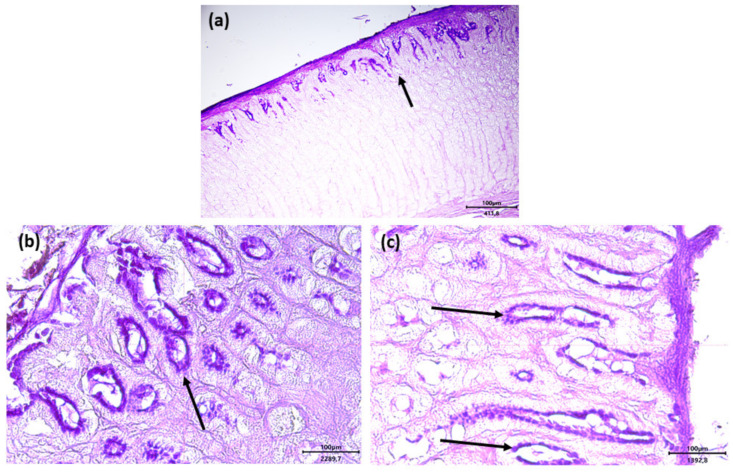
Fundic mucosa of the stomach, AB/PAS staining. (**a**), (**b**), and (**c**) samples indicate control, and groups supplemented with flaxseed and fish oil, respectively. Several mucous cells are detected containing mainly acidic (blue-stained, arrows) glycoconjugates. Proper scale bar is located in each figure. For the interpretation of the arrow and the asterisk see the text.

Alcian blue-periodic acid–Schiff (AB-PAS) histochemical staining showed that intestinal goblet cells contained both neutral (pink color) and acid glycoconjugates (blue color). Acid glycoconjugates appeared to be predominant especially in the treated groups (Figure 8a–c for control, F, and O groups, respectively, with arrows showing acid glycoconjugates).

## 3. Discussion

This study showed, for the first time, that n-3 PUFAs-enriched diets influence bacterial richness and diversity of the rabbit’s gut microbiota. Changes in microbial community composition and diversity, involving Firmicutes/Bacteroidetes ratio and *Lachnospiraceae* taxa, may contribute to the beneficial effects of n-3 PUFAs. Some differences were also found at the histological level, mainly affecting the stomach mucosa, suggesting that the supplementation dose should be optimized.

Regardless of the nutritional treatment, the main taxa detected were in accordance with previous studies evaluating the gut microbiota of both domesticated and wild rabbits [5,53,54]. The most abundant phyla were Firmicutes and Bacteroidetes. Firmicutes were, however, unaffected by nutritional treatment. Previous studies have evaluated the effects of fish oil supplementation on the fecal microbiota of mice, finding contrasting results. Cui et al. [13] showed that fish oil supplementation for 12 weeks reduced the relative abundance of Firmicutes, while Yu et al. [55] found a significant increase in this phylum after 15 days of supplementation in fecal samples of mice. A higher relative abundance of Bacteroidetes was instead found in fish oil-supplemented rabbits compared to the control group. This result was expected, as the association between dietary n-3 PUFA intake and Bacteroidetes increase has already been demonstrated in humans and mice [13,42,56]. This is extremely interesting, considering the Bacteroidetes’ positive role in modulating health status, in particular, in the context of diseases such as obesity [57]. In addition, the n-3 PUFAs supplementation changed the Epsilonbacteraeota at phylum, class, order, family, and genus level (i.e., Campylobacteria, Campylobacterales, *Campylobacteraceae* and *Campylobacter*). Their proportions within the microbial population were, however, negligible.

Regardless of nutritional groups, the prevailing classes were Bacilli, Bacteroidia, and Clostridia, and the same pattern was followed at the level of order. Interestingly, Bacilli (and also *Bacillales*) were more abundant in control than in treated groups. The same result was found in other studies conducted on mice fecal microbiota, where diets enriched with n-3 PUFA significantly lowered the Bacilli relative abundance [13,48,58]. In humans, this reduction has been associated with the consumption of a Mediterranean diet, rich in fish and seafood [42].

The dominant families were representative of the dominant orders and classes, with *Ruminococcaceae* and *Lachnospiraceae* as the main families. This partially confirms previous reports on rabbit gastrointestinal microbiota [5,53]. Importantly, the *Lachnospiraceae* family was higher in rabbits receiving n-3 PUFA supplementation in all gastrointestinal tracts. Moreover, at the genus level, a member of the family *Lachnospiraceae* showed significantly higher relative abundance in F and O groups than the C group, especially at the foregut level. These findings are in full agreement with previous studies highlighting the positive correlation between n-3 PUFAs intake, including both fish oil and flaxseed supplementations, and *Lachnospiraceae* abundance [39,42,44,49]. *Lachnospiraceae* is associated with increased production of short-chain fatty acid (SCFA) butyrate, which has beneficial effects thanks to its anti-inflammatory and antiatherosclerotic properties [39,42].

The importance of F/B ratio on several metabolic syndromes has been widely demonstrated both in humans and experimental animals [22,42,48]. It is a useful indicator of the homeostasis of the gastrointestinal microbiota and, in particular, high levels of this ratio have been associated with obesity [48]. Regardless of the gastrointestinal tract, the F/B ratio decreased in the PUFA supplemented groups. These findings fully agreed with previous studies carried out both in humans and laboratory animals [13,39,42]. It also suggests that changes in F/B ratio could mediate the beneficial effects of foods rich in n-3 PUFA [39,48]. In our study, this difference was not statistically significant when stratifying for the digestive tract. This could be probably ascribed to the high individual variability and limited sample size.

As expected, the alpha diversity results showed a higher richness and diversity in bacterial composition in the hindgut compared to the foregut, independently from the group. These results are in agreement with another study performed in rabbits [5], and can be explained by the physiological fermentative activity typical of colon and cecum tracts, which increases the microbial densities and also diversity at this level [21]. Flaxseed supplementation caused higher microbial diversity in the stomach compared with the C group; these findings can be related to the high protein contents of these seeds (10–30%), which can induce proliferation of the proteolytic bacterial population [45]. The O group, instead, showed higher microbial richness in the ileum compared to the C group. In general, the increase in the alpha diversity has beneficial effects by reducing the risk of dysbiosis, guaranteeing the complexity of the microbial ecosystem, which is fundamental for gut health [59]. These positive results are in agreement with what already shown in other studies examining the effect of both fish oil and flaxseed supplemented diets on caecal content and feces’ alpha diversity in mice [13,60]. Conversely, a reduction in alpha diversity was found in the colon of the O group. These results also seemed contradictory because not all indices were significant and one of them showed higher values than the C group. Similarly, the beta diversity results stratified by gastrointestinal tract seem inconsistent, as only a cluster at the stomach level was found. We can speculate that the small sample size could lead to a bias for these results. Microbiota composition in mice indeed showed well-defined clusters after fish oil and flaxseed supplementation [13,60].

Results about cecal bacterial fermentations indicate that both n*-3* PUFA-enriched diets did not affect lactic acid production, whereas flaxseed supplementation decreased ammonia fermentation. Ammonia is one of the microbial products that is known to have negative health effects in animals, which is excreted in large quantities, especially in intensive production [61,62]. Results regarding the F group indicated a good state of health of the animals, in accordance with Fraga [63], which indicated a range of 4.5–6.0 mmol/kg as adequate for protein microbial synthesis in the rabbit.

Surprisingly, the results concerning the histological analysis of the samples of the stomach collected by both F and O groups showed mild signs of hemorrhagic infiltration and vascular ectasia in the deepest part of the gastric mucosa compared to the control group. The same samples presented, however, a normal structural organization of the organ and no damage in the epithelium. Moreover, the samples obtained from the intestinal tracts did not show any kind of lesions in all the experimental groups. To date, there are no studies that report the same histological signs in the stomach of rabbits; there is indeed evidence that n-3 PUFA contained in fish oil does not induce negative effects on the histological structure of the liver and kidney [64]. At common dosage, n-3 fatty acids exert a plethora of physiological effects including lipid blood lowering, reduction in inflammatory indices, immunomodulation, and anti-thrombotic effects [65]. Despite their widespread use and beneficial actions, fish oil and flaxseed could determine some side effects at high dosage such hyperglycemia [66], bleeding [67], reduced blood pressure [68], diarrhea [69,70], acid reflux, and heartburn [71] in humans. The hemorrhagic infiltration of the lamina propria of the stomach mucosa could be due to the anti-thrombotic effect of n-3 PUFAs included in flaxseed and fish oil. It was reported that the consumption of large amounts of fish oil can inhibit blood clotting, increasing the risk of bleeding in humans. McEwen et al. [67] found that daily supplementation with 640 mg of fish oil for four weeks strongly reduced blood clotting in healthy adults. Additionally, another study reported that a high dose of fish oil consumption can increase the risk of nosebleeds as a side effect in adolescents [72]. Moreover, the apical ectatic vessels of the lamina propria found in the gastric samples collected from rabbits belonging to groups F and O could be explained by the effects of n-3 PUFAs on the endothelial function and vascular tone [73]. There is evidence that PUFAs are involved in the signaling pathways that both directly and indirectly induce vasodilation by the production of several mediators such as eicosanoids, cycloxygenase, and cytochrome P450 epoxygenase metabolites [74,75]. In the absence of other data of rabbit, it could be speculated that the hemorrhagic infiltrate and vascular ectasia found in the gastric samples is probably due to the high dosage of n-3 fatty acids included in the diets. Another possible explanation of the presence of the lesions in the stomach and not in the intestine could be that, in the latter, the n-3 PUFAs concentrations are lower as a consequence of the lipidic digestion and absorption as well as to their dilution with bile, pancreatic, and intestinal secretions. However, it must be highlighted that gastric lesions did not influence the health status as well as the growing rate of the rabbits fed with flaxseed and fish oil.

To the best of our knowledge, this is the first study that shows the effect of dietary supplementation with flaxseed and fish oil on the gut microbiota composition of rabbits, albeit on a limited number of animals. These preliminary results could represent the starting point for future research to evaluate whether these changes in bacterial populations may also modify the cecal fermentative production of volatile fatty acids. The relationship and the connection pathways between the changes in the gut microbiota and the efficiency of the immune system of the host could be investigated. Moreover, bioactive components of flaxseed and fish oil other than PUFAs could intervene in this relationship, and further research should focus on their role in gut microbiota changes. For example, flaxseed contains phytochemicals, such as isoflavones and lignans [34,76], which could influence the microbial flora as well as the reproductive sphere and the health of the rabbit. It is also important to note that extruded flaxseeds were used in the present study in order to decrease anti-nutritional factors such as tannins. Research should be addressed to study the adequate dosage usable to avoid the onset of possible side effects in rabbits. The supplementation of the diet with nutraceutical substances (flaxseed and fish oil) and the consequent changes in the gut microbiota may represent an innovative strategy to prevent and treat several gastrointestinal disorders as well as to increase the productive performance and welfare of the rabbit.

## 4. Materials and Methods

### 4.1. Animals and Samples Collection

The rabbits were reared in the experimental facility of the Department of Molecular and Developmental Medicine of University of Siena. At weaning, fifteen New Zealand White male rabbits were divided into three groups (*n* = 5/group) and fed three different diets (Table 3) until slaughter (90 days of age): control group (C), fed with a standard diet, flaxseed group (F), fed standard diet supplemented with 10% of extruded flaxseed, and fish oil group (O), fed standard diet supplemented with 3.5% of fish oil (Nordic Naturals Omega-3^®^, Watsonville, CA, USA). These diets have previously been used [33] and their fatty acid profile is shown in Table S6. For the entire trial, all diets and water were provided ad libitum. 

The rabbits were kept in a controlled environment for temperature (18–21 °C), relative humidity (60%), and light (16 h of day) in compliance with Directive 2010/63/EU related to the protection of animals kept for breeding purposes.

At the end of the trial, the animals were slaughtered in an authorized slaughterhouse, and the stunning (electrical), bleeding, and skinning of the animals followed the European Union regulations, specifically the Council Regulation No 1099/2009 on the protection of animals at the time of the slaughter. The average body weights (± standard error) at weaning were 857 ± 105 g, 879 ± 115 g, and 898 ± 125, whereas at slaughtering, they were 2310 ± 82 g, 2410 ± 120 g, and 2375 ± 55 g in C, F, and O groups, respectively.

The gastrointestinal tract was immediately removed from each animal. The luminal contents of the stomach, duodenum, jejunum, ileum, caecum, and colon were individually collected in a sterile 15 mL tube and stored in sterile tubes at −80 °C until analysis.

### 4.2. Microbiota Evaluation

#### 4.2.1. DNA Extraction

DNA was extracted from each fecal sample while using a QIAamp PowerFecal Pro DNA Kit (Qiagen, Hilden, Germany), according to the manufacturer’s protocol. DNA quality and quantity were assessed using a NanoDrop ND-1000 spectrophotometer (NanoDrop Technologies, Wilmington, DE, USA). The isolated DNA was then stored at −20 °C until use.

#### 4.2.2. Next Generation 16S rRNA Gene Sequencing

Bacterial DNA was amplified using the primers that were described in literature [77], which target the V3-V4 hypervariable regions of the 16S rRNA gene. All the PCR amplifications were performed in 25 µL volumes per sample. A total of 12.5 µL of KAPA HIFI Master Mix 2× (Kapa Biosystems, Inc., Wilmington, MA, USA) and 0.2 µL of each primer (100 µM) were added to 2 µL of genomic DNA (5 ng/µL). The blank controls (no DNA template added to the reaction) were also performed. A first amplification step was performed in an Applied Biosystem 2700 thermal cycler (ThermoFisher Scientific, Waltham, MA USA). The samples were denatured at 95 °C for 3 min, followed by 25 cycles with a denaturing step at 98 °C for 30 s, annealing at 56 °C for 1 min, and extension at 72 °C for 1 min, with a final extension at 72 °C for 7 min. The amplicons were then cleaned with Agencourt AMPure XP (Beckman, Coulter Brea, CA, USA), and libraries were prepared following the 16S Metagenomic Sequencing Library Preparation Protocol (Illumina, San Diego, CA, USA). The libraries obtained were quantified using real-time PCR with KAPA Library Quantification Kits (Kapa Biosystems, Inc., Wilmington, MA, USA), pooled in equimolar proportion and then sequenced in one MiSeq (Illumina, San Diego, CA, USA) run with 2 × 250-base paired-end reads.

#### 4.2.3. Sequence Analysis

Demultiplexed paired-end reads from 16S rRNA-gene sequencing were first checked for quality while using FastQC [78] for an initial assessment. The forward and reverse paired-end reads were joined into single reads while using the C++ program SeqPrep [79].

After joining, the reads were filtered for quality based on: (i) maximum three consecutive low-quality base calls (Phred <19) allowed; (ii) the fraction of consecutive high-quality base calls (Phred >19) in a read over total read length ≥0.75; and (iii) no “N”-labeled bases (missing/uncalled) allowed. Reads that did not match all the above criteria were filtered out. All the remaining reads were combined in a single FASTA file for the identification and quantification of OTUs (operational taxonomic units). The reads were aligned against the SILVA closed reference sequence collection release 132, with 97% cluster identity [80,81] applying the Cd-hit clustering algorithm [82]. A pre-defined taxonomy map of reference sequences was then used for taxonomic identification along the main taxa ranks down to the genus level (domain, phylum, class, order, family, genus). By counting the abundance of each OTU, the OTU table was created and then grouped at each phylogenetic level. OTUs with total counts lower than 50 in fewer than 5 samples were filtered out. All of the above steps, except the FastQC reads quality check, were performed with the QIIME 1.9 open-source bioinformatics pipeline for microbiome analysis [83]. The command lines and parameters that were used to process 16S rRNA gene sequence data are detailed in Biscarini et al. [84].

#### 4.2.4. Alfa and Beta Diversity Indices

The microbial diversity of the different niches of the rabbit gastrointestinal tract was assessed within-(alpha diversity) and across-(beta diversity) samples. All the indices (alpha and beta diversity) were estimated from the OTU table (at the OTU level), filtered for OTUs with more than 50 total counts distributed in at least five samples. Besides the number of observed OTUs directly counted from the OTU table, within-sample microbial richness, diversity, and evenness were estimated using the following indices: Chao1 and ACE (abundance-based coverage estimator) for richness, Shannon, Simpson, and Fisher’s alpha for diversity [85,86], and Simpson E and Pielou’s J (Shannon’s evenness) for evenness [87]. The across-sample microbiota diversity was quantified by calculating Bray-Curtis dissimilarities [88]. Prior to the calculation of alpha and beta diversity metrics, the OTU counts were normalized for uneven sequencing depth by cumulative sum scaling CSS [89]. Details on the calculation of the mentioned alpha- and beta-diversity indices can be found in Biscarini et al. [84].

#### 4.2.5. Lactic Acid and Ammonia Quantification

For the analysis of bacterial metabolites (lactic acid and ammonia), 1 g of cecal content was diluted in 1 mL of 1 M perchloric acid and 8 mL of distilled water. After homogenization, tubes were centrifuged for 10 min at 5000 rpm, and the supernatant was transferred to 2 mL eppendorf and frozen at −20 °C until metabolites quantification.

The spectrophotometric method for biological fluids was used for lactic acid determination according to Pryce et al. [90]. Ammonia concentration was detected in accordance with Patton et al. [91]. The spectrophotometer (Shimadzu Corporation UV-2550, Kyoto, Japan) was set at 565 nm and 660 nm respectively. All chemicals were purchased from Sigma Chemical Co (St Louis, MO, USA).

#### 4.2.6. Histology and Histochemistry

Samples of stomach body (fundic mucosa), proximal duodenum, jejunum, and ileum of 5 animals for each experimental group were collected, fixed in 10% formaldehyde in 0.01 M phosphate-buffered saline (PBS) pH 7.4 for 24 h at 4 °C, dehydrated, cleared with xylene, and embedded in paraffin. Microtome sections (4 µm thick) were stained with a) Hematoxylin–Eosin (HE) staining to establish structural details and b) Alcian blue 8GX- pH 2.5-periodic acid-Schiff (AB-PAS), which revealed neutral (PAS-reactive, purple stained) and acid (AB-reactive, azure stained) glycoconjugates. This histochemical reaction selectively evidenced both the intestinal mucous cells and the adherent mucous gel, which is synthesized by the mucous cells and lies upon the mucosal epithelial layer.

## 5. Conclusions

This study provides novel evidence that the dietary supplementation of flaxseed and fish oil can have a beneficial impact on the composition of gut microbiota and cecal fermentation in rabbits. In particular, n-3 PUFAs supplementation increased some beneficial populations such as Bacteroidetes and *Lachnospiraceae* while reducing Bacilli and the Firmicutes/Bacteroidetes ratio, usually associated with metabolic disease, opening interesting scenarios about the reduction in the use of antibiotics in rabbit farms. Further studies are warranted to investigate the mechanism of action of n-3 PUFAs and other bioactive components included in flaxseed and fish oil, which favor the modification of the gut microbiota. Targeting the gut microbiota populations composition and its metabolic activity with the diet could be a promising strategy, not only to maintain intestinal homeostasis, but also to control the immune as well as inflammatory response, and consequently, the incidence of gastrointestinal disorders of the rabbit. The appropriate quantity of flaxseed and fish oil to introduce with the diet to favor the right prebiotic action should be, however, optimized.

## Figures and Tables

**Table 1:** Values for the alpha diversity indices in the three experimental groups (control, and supplemented with flaxseed (Flaxseed) and fish oil (Fish oil); *n* = 5/group) along the rabbit’s gastrointestinal tract.

Group	Gastrointestinal Tract	Chao1	Ace	Fisher Alpha	Observed OTUS	Shannon	Simpson	Equitability	Simpson e
Control	Stomach	233.250	233.250	101.578	233.25	7.646	0.994	0.980	0.797
Control	Duodenum	344.533	334.476	147.037	316.00	8.043	0.996	0.973	0.737
Control	Jejunum	258.000	258.000	112.640	258.00	7.772	0.995	0.971	0.725
Control	Ileum	325.317	312.777	132.726	293.25	7.941	0.995	0.971	0.729
Control	Caecum	814.740	787.556	328.752	638.75	8.970	0.998	0.963	0.649
Control	Colon	781.027	779.864	329.898	661.00	9.033	0.998	0.964	0.661
Flaxseed	Stomach	654.676	644.354	274.021	545.00	8.755	0.997	0.964	0.665
Flaxseed	Duodenum	415.105	404.532	173.917	367.25	8.214	0.996	0.967	0.687
Flaxseed	Jejunum	465.343	456.567	203.200	431.50	8.500	0.997	0.972	0.713
Flaxseed	Ileum	366.754	357.585	154.462	335.00	8.141	0.996	0.972	0.727
Flaxseed	Caecum	759.350	761.723	316.750	609.25	8.874	0.997	0.961	0.640
Flaxseed	Colon	693.944	713.153	310.682	613.75	8.937	0.998	0.965	0.667
Fish oil	Stomach	470.384	463.068	193.549	388.25	8.127	0.995	0.971	0.718
Fish oil	Duodenum	326.839	311.604	134.043	294.00	7.873	0.995	0.970	0.712
Fish oil	Jejunum	534.375	541.931	230.253	461.50	8.336	0.996	0.969	0.694
Fish oil	Ileum	515.576	502.106	225.529	438.00	8.442	0.996	0.966	0.672
Fish oil	Caecum	865.822	844.204	350.033	677.25	9.030	0.998	0.962	0.642
Fish oil	Colon	658.432	686.182	300.589	593.25	8.890	0.997	0.965	0.665

**Table 2:** Caecum lactic acid and ammonia quantification in the three experimental groups, Control (*n* = 5), Flaxseed (*n* = 5), and Fish oil (*n* = 5). Results are expressed as mean ± standard deviation. * *p* < 0.05 vs. control group.

Group	Lactate (mmol/kg)	Ammonia (mmol/kg)
Control	7.92 ± 3.43	8.03 ± 2.34
Flaxseed	4.74 ± 1.66	5.01 * ± 1.42
Fish Oil	5.26 ± 2.48	8.98 ± 2.44

**Table 3:** Formulation and chemical composition (on fresh matter) of control and n-3-enriched diets.

Ingredients (g/kg)	Control	Flaxseed	Fish Oil
Dehydrated alfalfa meal	300	380	380
Soybean meal 44%	150	110	150
Barley meal	410	310	335
Wheat bran	52	52	52
Soybean oil	30	-	-
Extruded flaxseed	-	100	-
Fish oil *	-	-	35
Beet molasses	20	10	10
Calcium carbonate	7	7	7
Calcium diphosphate	13.5	13.5	13.5
Salt	7	7	7
DL-methionine	0.5	0.5	0.5
Vitamin-mineral premix **	10	10	10
Crude protein	175	174	175
Ether extract	480	472	425
Crude Fiber	124	137	130
Ash	89	84	90

∗ Nordic Naturals Omega-3^®^ = purified deep sea fish oil (from anchovies and sardines) containing EPA—330 mg/100 g, DHA—220 mg/100 g, and other n-3 LC PUFA—140 mg/100 g + α-tocopherol for preservation. Per kg diet: vitamin A—11.000 IU; vitamin D3—2000 IU; vitamin B1—2.5 mg; vitamin B2—4 mg; vitamin B6—1.25 mg; vitamin B12—0.01 mg; alpha-tocopheryl acetate—200 mg; biotine—0.06 mg; vitamin K—2.5 mg; niacin—15 mg; folic acid—0.30 mg; D-pantothenic acid—10 mg; choline—600 mg; Mn—60 mg; Fe—50 mg; Zn—15 mg; I—0.5 mg; Co—0.5 mg. ** Per kg diet: vitamin A 11,000 IU; vitamin D3 2000 IU; vitamin B1 2.5 mg; vitamin B2 4 mg; vitamin B6 1.25 mg; vitamin B12 0.01 mg; alpha-tocopherol acetate 50 mg; biotine 0.06 mg; vitamin K 2.5 mg; niacin 15 mg; folic acid 0.30 mg; D-pantothenic acid 10 mg; choline 600 mg; Mn 60 mg; Fe 50 mg; Zn 15 mg; I 0.5 mg; Co 0.5 mg.

## Data Availability

The data presented in this study are available in the article and Supplementary Materials. Further information is available upon request from the corresponding author.

## Supplementary Materials

The following are available online at https://www.mdpi.com/article/10.3390/antibiotics11020227/s1, Table S1: Significantly different taxa at the OTU level, Table S2: Abundance of the significantly different taxa according with group and gastrointestinal tract, Table S3: Between-treatment differences along the rabbit’s gastrointestinal tract, Table S4: Coefficient estimates and *p*-values for the F/B ratio in treatments vs. control (benchmark), from a linear model, which included the effects of tissue and treatment. Table S5: Significance of differences between treatments in terms of Bray–Curtis distances for each section of the rabbit’s digestive tract. Table S6. Fatty acids profile as of control and n-3 enriched diets. Figure S1: Boxplot of the alpha diversity indices in the three groups in the digestive system of rabbits.
