# Supplementary material for: Could Dietary Supplementation with Different Sources of N-3 Polyunsaturated Fatty Acids Modify the Rabbit Gut Microbiota?
Source: Antibiotics (Basel). 2022 Feb 10;11(2):227. doi: 10.3390/antibiotics11020227 (PMC8868100; doi:10.3390/antibiotics11020227)
Supplement: Supplementary file 1 [file antibiotics-11-00227-s001.zip › antibiotics-1560063-supplementary.pdf]

Supplementary materials

Table S1. Significantly different taxa at the OTU level. OTUs are grouped per taxonomic level (from phylum to genus). Groups (n=5/group): control and enriched diets supplemented with flaxseed (flax) and fish oil (oil). P-values were obtained from a linear model that included the effects of treatment and tissue within rabbit.

Level	New taxa	Term	P-value
Phylum	Bacteroidetes	Treatment	0.025214434
Phylum	Epsilonbacteraeota	Treatment	0.001264694
Class	Bacteroidia	Treatment	0.025214434
Class	Campylobacteria	Treatment	0.001264694
Class	Negativicutes	Treatment	0.028893502
Order	Bacteroidales	Treatment	0.024852797
Order	Campylobacterales	Treatment	0.001264694
Order	Rhodobacterales	Treatment	0.017630764
Order	Selenomonadales	Treatment	0.028893502
Family	Acidaminococcaceae	Treatment	0.028893502
Family	Arachis duranensis	Treatment	0.045780306
Family	Campylobacteraceae	Treatment	0.012860544
Family	Gut metagenome	Treatment	0.041133323
Family	Marinifilaceae	Treatment	0.049934817
Family	Rhodobacteraceae	Treatment	0.017630764
Family	Victivallaceae	Treatment	0.041557662
Genus	[acetivibrio] ethanolgignens group	Treatment	0.022229071
Genus	[eubacterium] coprostanoligenes group	Treatment	0.035170353
Genus	[eubacterium] xylanophilum group	Treatment	0.024695621
Genus	Acidovorax	Treatment	0.001683302
Genus	Alistipes	Treatment	0.049600553
Genus	Brevibacillus	Treatment	0.019368428
Genus	Campylobacter	Treatment	0.012860544
Genus	Falsirhodobacter	Treatment	0.017630764
Genus	Lachnospiraceae ucg-001	Treatment	0.000672891
Genus	Mesorhizobium	Treatment	0.023102407
Genus	Phascolarctobacterium	Treatment	0.028893502
Genus	Ruminiclostridium 1	Treatment	0.040451103
Genus	Victivallis	Treatment	0.041557662

Table S2. Abundance of the significantly different taxa according with group and gastrointestinal tract.

Level	Taxon	Treatment	Stomach	Duode-num	Jejunum	Ileum	Caecum	Colon
Phylum	Bacteroidetes	Control	128.96	162.25	126.77	180.53	377.32	398.90
Phylum	Bacteroidetes	Flaxseed	366.17	243.81	234.20	211.49	389.40	375.67
Phylum	Bacteroidetes	Fish oil	242.78	207.32	289.52	309.95	428.53	411.32
Phylum	Epsilonbacteraeota	Control	2.10	1.92	0.72	2.68	8.06	8.24
Phylum	Epsilonbacteraeota	Flaxseed	7.36	4.98	3.22	3.68	8.63	7.84
Phylum	Epsilonbacteraeota	Fish oil	8.35	5.37	5.24	6.30	13.26	12.92
Class	Bacteroidia	Control	128.96	162.25	126.77	180.53	377.32	398.90
Class	Bacteroidia	Flaxseed	366.17	243.81	234.20	211.49	389.40	375.67
Class	Bacteroidia	Fish oil	242.78	207.32	289.52	309.95	428.53	411.32
Class	Campylobacteria	Control	2.10	1.92	0.72	2.68	8.06	8.24
Class	Campylobacteria	Flaxseed	7.36	4.98	3.22	3.68	8.63	7.84
Class	Campylobacteria	Fish oil	8.35	5.37	5.24	6.30	13.26	12.92
Class	Negativicutes	Control	0.00	1.49	0.00	0.00	0.34	0.00
Class	Negativicutes	Flaxseed	6.06	4.07	2.29	4.64	6.80	6.84
Class	Negativicutes	Fish oil	0.49	1.64	0.94	2.29	2.16	1.54
Order	Bacteroidales	Control	125.91	161.09	125.17	176.02	374.67	396.29
Order	Bacteroidales	Flaxseed	359.87	240.31	230.67	209.70	386.98	372.66
Order	Bacteroidales	Fish oil	237.97	204.57	289.26	308.66	427.85	410.97
Order	Campylobacterales	Control	2.10	1.92	0.72	2.68	8.06	8.24
Order	Campylobacterales	Flaxseed	7.36	4.98	3.22	3.68	8.63	7.84
Order	Campylobacterales	Fish oil	8.35	5.37	5.24	6.30	13.26	12.92
Order	Rhodobacterales	Control	0.00	0.00	0.00	0.00	0.00	0.00
Order	Rhodobacterales	Flaxseed	0.00	0.84	0.51	1.42	0.00	0.00
Order	Rhodobacterales	Fish oil	0.00	0.00	0.00	0.00	0.00	0.00
Order	Selenomonadales	Control	0.00	1.49	0.00	0.00	0.34	0.00
Order	Selenomonadales	Flaxseed	6.06	4.07	2.29	4.64	6.80	6.84
Order	Selenomonadales	Fish oil	0.49	1.64	0.94	2.29	2.16	1.54
Family	Acidaminococcaceae	Control	0.00	1.49	0.00	0.00	0.34	0.00
Family	Acidaminococcaceae	Flaxseed	6.06	4.07	2.29	4.64	6.80	6.84
Family	Acidaminococcaceae	Fish oil	0.49	1.64	0.94	2.29	2.16	1.54
Family	Arachis duranensis	Control	0.00	0.00	0.00	0.00	0.00	0.00
Family	Arachis duranensis	Flaxseed	0.00	0.00	0.00	0.00	0.00	0.00
Family	Arachis duranensis	Fish oil	2.10	0.00	0.94	0.00	0.00	0.00
Family	Campylobacteraceae	Control	2.10	1.92	0.72	1.73	8.06	8.24
Family	Campylobacteraceae	Flaxseed	7.36	4.30	2.71	3.68	8.63	7.84
Family	Campylobacteraceae	Fish oil	4.09	5.37	5.24	3.97	12.52	12.92
Family	Gut metagenome	Control	0.00	0.00	0.00	0.00	0.00	0.00
Family	Gut metagenome	Flaxseed	2.55	1.18	2.43	0.00	1.39	0.36
Family	Gut metagenome	Fish oil	0.00	0.65	0.00	0.00	0.00	0.00
Family	Marinifilaceae	Control	10.28	12.88	6.28	12.08	33.37	32.03
Family	Marinifilaceae	Flaxseed	31.98	15.53	16.79	18.05	31.01	28.36
Family	Marinifilaceae	Fish oil	26.80	14.23	21.19	23.02	39.55	34.78

Family	Rhodobacteraceae	Control	0.00	0.00	0.00	0.00	0.00	0.00
Family	Rhodobacteraceae	Flaxseed	0.00	0.84	0.51	1.42	0.00	0.00
Family	Rhodobacteraceae	Fish oil	0.00	0.00	0.00	0.00	0.00	0.00
Family	Victivallaceae	Control	0.72	0.63	0.53	0.91	3.00	2.76
Family	Victivallaceae	Flaxseed	5.16	6.20	6.73	3.47	3.01	3.51
Family	Victivallaceae	Fish oil	0.76	3.89	1.78	1.70	2.38	1.08
Genus	[acetivibrio] ethanolgignens group	Control	0.78	2.24	0.69	2.37	3.19	3.11
Genus	[acetivibrio] ethanolgignens group	Flaxseed	3.59	3.11	3.73	1.67	2.82	3.65
Genus	[acetivibrio] ethanolgignens group	Fish oil	1.22	1.33	2.54	2.08	3.12	2.37
Genus	[eubacterium] coprostanoligenes group	Control	5.00	7.03	5.43	5.85	19.62	22.51
Genus	[eubacterium] coprostanoligenes group	Flaxseed	16.33	7.86	5.07	5.56	16.48	17.11
Genus	[eubacterium] coprostanoligenes group	Fish oil	18.02	13.29	21.98	13.20	29.22	28.66
Genus	[eubacterium] xylanophilum group	Control	3.37	6.00	2.18	4.30	12.31	16.54
Genus	[eubacterium] xylanophilum group	Flaxseed	9.61	7.71	13.47	4.03	12.24	12.85
Genus	[eubacterium] xylanophilum group	Fish oil	7.93	11.41	13.22	11.40	13.64	13.48
Genus	Acidovorax	Control	0.00	0.00	0.00	0.00	0.00	0.00
Genus	Acidovorax	Flaxseed	0.00	1.12	0.85	2.13	0.00	0.00
Genus	Acidovorax	Fish oil	0.00	0.00	0.00	0.00	0.00	0.00
Genus	Alistipes	Control	25.13	34.56	16.68	32.65	80.61	86.72
Genus	Alistipes	Flaxseed	83.00	50.06	47.67	27.43	83.46	86.03
Genus	Alistipes	Fish oil	47.79	44.95	61.74	67.82	90.20	91.06
Genus	Brevibacillus	Control	5.92	2.41	2.43	0.67	0.00	0.00
Genus	Brevibacillus	Flaxseed	0.63	0.00	0.00	0.97	0.00	0.00
Genus	Brevibacillus	Fish oil	2.27	1.30	2.08	2.36	0.40	0.00
Genus	Campylobacter	Control	2.10	1.92	0.72	1.73	8.06	8.24
Genus	Campylobacter	Flaxseed	7.36	4.30	2.71	3.68	8.63	7.84
Genus	Campylobacter	Fish oil	4.09	5.37	5.24	3.97	12.52	12.92
Genus	Falsirhodobacter	Control	0.00	0.00	0.00	0.00	0.00	0.00
Genus	Falsirhodobacter	Flaxseed	0.00	0.84	0.51	1.42	0.00	0.00
Genus	Falsirhodobacter	Fish oil	0.00	0.00	0.00	0.00	0.00	0.00
Genus	Lachnospiraceae ucg-001	Control	7.55	4.95	3.35	4.28	7.04	4.33
Genus	Lachnospiraceae ucg-001	Flaxseed	10.53	4.14	6.37	3.05	8.89	6.80
Genus	Lachnospiraceae ucg-001	Fish oil	0.79	0.65	1.19	2.35	4.85	2.75
Genus	Mesorhizobium	Control	0.00	0.00	0.00	0.00	0.00	0.00
Genus	Mesorhizobium	Flaxseed	0.00	0.38	2.20	2.09	0.00	0.00
Genus	Mesorhizobium	Fish oil	0.30	0.60	0.77	0.00	0.00	0.00
Genus	Phascolarctobacterium	Control	0.00	1.49	0.00	0.00	0.34	0.00
Genus	Phascolarctobacterium	Flaxseed	6.06	4.07	2.29	4.64	6.80	6.84
Genus	Phascolarctobacterium	Fish oil	0.49	1.64	0.94	2.29	2.16	1.54
Genus	Ruminiclostridium 1	Control	1.59	1.15	0.00	0.00	7.49	7.94
Genus	Ruminiclostridium 1	Flaxseed	4.07	2.25	2.64	1.93	6.57	6.46

Genus	Ruminiclostridium 1	Fish oil	3.11	2.27	3.77	2.48	12.77	11.44
Genus	Victivallis	Control	0.72	0.63	0.53	0.91	3.00	2.76
Genus	Victivallis	Flaxseed	5.16	6.20	6.73	3.47	3.01	3.51
Genus	Victivallis	Fish oil	0.76	3.89	1.78	1.70	2.38	1.08

Table S3. Between-treatment differences along the rabbit's gastrointestinal tract, for the significantly different taxa identified in Figure 3. Difference is marked by "1", lack of difference by "0". Between-treatment differences were evaluated by linear models of abundance as a function of treatments, run separately for each intestinal segment. Given that within-segment models were based on a smaller number of samples, p-values may differ from those in Figure 3.

Level	New taxa	Stomach	Duodenum	Jejunum	Ileum	Caecum	Colon
Class	Bacteroidia	1	0	0	1	0	0
Class	Campylobacteria	1	0	0	0	1	1
Class	Negativicutes	1	0	0	1	1	1
Family	Acidaminococcaceae	1	0	0	1	1	1
Family	Arachis duranensis	1	0	0	0	0	0
Family	Campylobacteraceae	1	0	0	0	1	1
Family	Gut metagenome	1	0	1	0	1	0
Family	Marinifilaceae	1	0	0	1	0	0
Family	Rhodobacteraceae	0	0	0	1	0	0
Family	Victivallaceae	1	1	1	0	0	0
Genus	[acetivibrio] ethanolgignens group	1	0	1	0	0	1
Genus	[eubacterium] coprostanoligenes group	0	0	1	0	1	0
Genus	[eubacterium] xylanophilum group	0	0	1	1	0	0
Genus	Acidovorax	0	1	0	1	0	0
Genus	Alistipes	1	0	0	1	0	0
Genus	Brevibacillus	1	0	0	0	0	0
Genus	Campylobacter	1	0	0	0	1	1
Genus	Falsirhodobacter	0	0	0	1	0	0
Genus	Lachnospiraceae ucg-001	0	1	1	0	0	1
Genus	Mesorhizobium	0	0	1	1	0	0
Genus	Phascolarctobacterium	1	0	0	1	1	1
Genus	Ruminiclostridium 1	0	0	0	0	1	1
Genus	Victivallis	1	1	1	0	0	0
Order	Bacteroidales	1	0	0	1	0	0
Order	Campylobacterales	1	0	0	0	1	1
Order	Rhodobacterales	0	0	0	1	0	0
Order	Selenomonadales	1	0	0	1	1	1
Phylum	Bacteroidetes	1	0	0	1	0	0
Phylum	Epsilonbacteraeota	1	0	0	0	1	1

Table S4. Coefficient estimates and p-values for the F/B ratio in treatments vs control (benchmark), from a linear model which included the effects of tissue and treatment

Tissue	Term	Estimate	Std.error	Statistic	P.value
Stomach	Treatmentflax	-2.2538	1.1111	-2.0284	0.0731
Stomach	Treatmentoil	-2.4081	1.1111	-2.1673	0.0584
Duodenum	Treatmentflax	-2.1778	1.0302	-2.1140	0.0637
Duodenum	Treatmentoil	-2.1034	1.0302	-2.0418	0.0716
Jejunum	Treatmentflax	-0.1000	1.6258	-0.0615	0.9523
Jejunum	Treatmentoil	-1.8060	1.6258	-1.1108	0.2954
Ileum	Treatmentflax	-0.2373	0.6434	-0.3688	0.7219
Ileum	Treatmentoil	-1.4041	0.6950	-2.0204	0.0780
Caecum	Treatmentflax	-0.4003	0.3091	-1.2950	0.2276
Caecum	Treatmentoil	-0.4657	0.3091	-1.5065	0.1662
Colon	Treatmentflax	0.0167	0.4029	0.0415	0.9678
Colon	Treatmentoil	-0.8334	0.4029	-2.0687	0.0685

Table S5. Significance of differences between treatments in terms of Bray-Curtis distances for each section of the rabbit's digestive tract.

Tissue	Term	Df	Sums Of Sqs	Mean Sqs	F. Model	R2	Pr (>F)
Ileum	Treatment	2	0.2255021456	0.1127510728	0.8902435703	0.1820448322	0.707
Ileum	Residuals	8	1.013215498	0.1266519373		0.8179551678	
Ileum	Total	10	1.238717644			1	
Jejunum	Treatment1	2	0.3289109197	0.1644554598	1.230255812	0.2146947453	0.224
Jejunum	Residuals1	9	1.203082419	0.1336758244		0.7853052547	
Jejunum	Total1	11	1.531993339			1	
Duodenum	Treatment2	2	0.2372168078	0.1186084039	0.8930619657	0.1655946049	0.761
Duodenum	Residuals2	9	1.195298508	0.1328109453		0.8344053951	
Duodenum	Total2	11	1.432515316			1	
Colon	Treatment3	2	0.1333038994	0.06665194969	1.061601843	0.1908805904	0.255
Colon	Residuals3	9	0.5650588786	0.06278431985		0.8091194096	
Colon	Total3	11	0.698362778			1	
Caecum	Treatment4	2	0.1237146091	0.06185730456	1.045274793	0.1884982859	0.260
Caecum	Residuals4	9	0.5326022827	0.05917803141		0.8115017141	
Caecum	Total4	11	0.6563168918			1	
Stomach	Treatment5	2	0.5207751034	0.2603875517	2.00842282	0.3085882518	0.010
Stomach	Residuals5	9	1.166829983	0.1296477759		0.6914117482	
Stomach	Total5	11	1.687605087			1	

Table S6. Fatty acids profile (% of total fatty acids) as of control and n-3 enriched diets.

Fatty acids	**Control**	**Flax seed**	**Fish Oil**	Pooled SE
SFA	19.80	15.40	38.10	1.82
MUFA	17.40	15.80	14.50	0.87
PUFA	62.80	68.80	47.40_b	5.12
LA	50.45	22.30_a	20.50	2.11
ALA	11.15	45.80	18.50	1.42
LC PUFAn-3	-	-	10.50	1.00
EPA	-	-	3.50	0.21
DHA	-	-	4.20	0.28
n-6	51.45	22.80	21.00	2.35
n-3	11.35	46.00	26.40	1.55
n-6/n-3	4.53	0.50	0.80_a	0.01

Legend: SFA, saturated fatty acids; MUFA, monounsaturated fatty acids; PUFA, polyunsaturated fatty acids; LA, linoleic acid; ALA, α-linolenic acid; LC PUFA, Long Chain PUFA; EPA, eicosapentaenoic acid; DHA, docosahexaenoic acid.

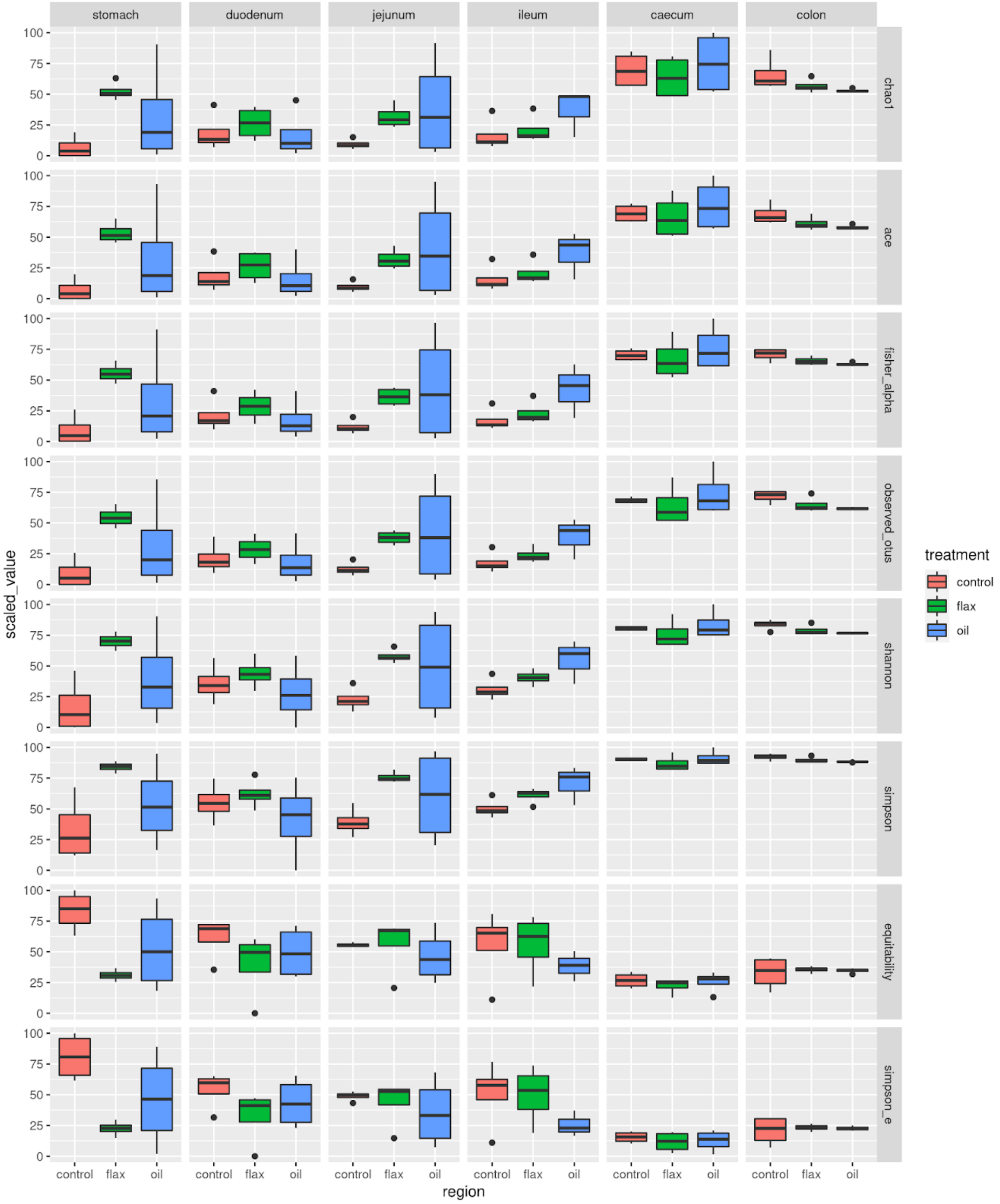

Figure S1. Boxplot of the alpha diversity indices in the three groups (red: control; gree: flax-supplemented diet; blue: oil-supplemented diet) in the digestive system of rabbits.
